# Multiple responses contribute to the enhanced drought tolerance of the autotetraploid *Ziziphus jujuba* Mill. var. *spinosa*

**DOI:** 10.1186/s13578-021-00633-1

**Published:** 2021-06-30

**Authors:** Meng Li, Chenxing Zhang, Lu Hou, Weicong Yang, Songshan Liu, Xiaoming Pang, Yingyue Li

**Affiliations:** 1 Beijing Advanced Innovation Center for Tree Breeding by Molecular Design, Beijing Forestry University, Beijing, 100083 China; 2 National Engineering Laboratory for Tree Breeding, Beijing Forestry University, Beijing, 100083 China; 3 College of Biological Sciences and Technology, Beijing Forestry University, Beijing, 100083 China

**Keywords:** Chinese jujube, Autotetraploid, Drought tolerance, Physiology, Comparative transcriptome

## Abstract

**Background:**

Polyploid plants often exhibit enhanced stress tolerance. The underlying physiological and molecular bases of such mechanisms remain elusive. Here, we characterized the drought tolerance of autotetraploid sour jujube at phenotypic, physiological and molecular levels.

**Results:**

The study findings showed that the autotetraploid sour jujube exhibited a superior drought tolerance and enhanced regrowth potential after dehydration in comparison with the diploid counterpart. Under drought stress, more differentially expressed genes (DEGs) were detected in autotetraploid sour jujube and the physiological responses gradually triggered important functions. Through GO enrichment analysis, many DEGs between the diploid and autotetraploid sour jujube after drought-stress exposure were annotated to the oxidation–reduction process, photosystem, DNA binding transcription factor activity and oxidoreductase activity. Six reactive oxygen species scavenging-related genes were specifically differentially expressed and the larger positive fold-changes of the DEGs involved in glutathione metabolism were detected in autotetraploid. Consistently, the lower O^2−^ level and malonaldehyde (MDA) content and higher antioxidant enzymes activity were detected in the autotetraploid under drought-stress conditions. In addition, DEGs in the autotetraploid after stress exposure were significantly enriched in anthocyanin biosynthesis, DNA replication, photosynthesis and plant hormone, including auxin, abscisic acid and gibberellin signal-transduction pathways. Under osmotic stress conditions, genes associated with the synthesis and transport of osmotic regulators including anthocyanin biosynthesis genes were differentially expressed, and the soluble sugar, soluble protein and proline contents were significantly higher in the autotetraploid. The higher chlorophyll content and DEGs enriched in photosynthesis suggest that the photosynthetic system in the autotetraploid was enhanced compared with diploid during drought stress. Moreover, several genes encoding transcription factors (TFs) including GRAS, Bhlh, MYB, WRKY and NAC were induced specifically or to higher levels in the autotetraploid under drought-stress conditions, and hub genes, *LOC107403632*, *LOC107422279*, *LOC107434947*, *LOC107412673* and *LOC107432609*, related to 18 up-regulated transcription factors in the autotetraploid compared with the diploid were identified.

**Conclusion:**

Taken together, multiple responses contribute to the enhanced drought tolerance of autotetraploid sour jujube. This study could provide an important basis for elucidating the mechanism of tolerance variation after the polyploidization of trees.

**Supplementary Information:**

The online version contains supplementary material available at 10.1186/s13578-021-00633-1.

## Background

As sessile organisms, plants are exposed to harsh environmental conditions, including low and high temperatures, drought and salinity. Notably, owing to rapid changes in the global climate, freshwater resources have been drastically reduced, and land droughts are gradually increasing worldwide [1–3]. The regions classified as arid in the world cover approximately 6.1 billion hectares, account for 41% of the land area, and an increase in the arid land area will have dangerous consequences on food security and meeting basic human needs [4]. Therefore, research on the cultivation of drought-resistant germplasms and their drought-resistance mechanisms have vital roles in improving crop productivity and quality.

Plants have gradually formed adaptively survival strategies to severe environmental conditions through long-term co-evolution and constant natural selection. Under drought-stress conditions, significant changes in cell physiology and biochemistry occur, including decreases in turgor pressure, changes in the plasma membrane composition and fluidity, and changes in cell solute concentration and protein–lipid interactions [5]. Large numbers of physiological and metabolic pathways undergo changes, including decreases in photosynthetic activity, accumulations of organic acids and osmotic regulators, changes in carbohydrate metabolism, increases in protein-protective complex synthesis, enhanced energy and lipid metabolism, and the removal of reactive oxygen species (ROS), to adapt to a drought environment [6, 7]. In addition, the sensing and inward transduction of the drought signals by the corresponding receptors on the cell membrane lead to gene expression and transcription factor regulation, which effect the expression of corresponding functional proteins.

Polyploidization is an effective way for plants to adapt to the environment, and polyploidy breeding plays an important role in agriculture and forestry. Polyploid organisms have more than two sets of chromosomes, and polyploids are divided into allopolyploids, having different species’ chromosomes, such as cotton (*Gossypium* L.) and wheat (*Triticum*), and autopolyploids, having the same species’ chromosomes, such as potato (*Solanun tuberosum* L.). The gene expression level is always altered during polyploidization. Jackson and Chen suggested three possible mechanisms that mediate gene expression changes in polyploids, dosage effects of doubled genes, interactions regulated by modifications, and rapidly genetic and epigenetic modifications and changes [8]. These effects cause polyploid plants to exhibit different phenotypes, photosynthetic and physiological habits and metabolite contents than their diploid ancestors [9–12]. Examples include the dwarf in tetraploid apple [13], an increased photosynthetic rate in autotetraploid Chinese woad [14], and changes in auxin (Aux), abscisic acid (ABA) and other hormone contents in the leaves of autotetraploid pak choi [15]. Moreover, polyploidization enhances the plant’s adaptability to extreme environmental conditions [16]. For example, polyploidy in *Populus* [17], *Robinia pseudoacacia* [18], *Citrus* [19], *Paulownia* [20, 21] and other plant species enhances stress resistance compared with their diploid ancestors. However, how polyploidy or wide-genome doubling events change resistance traits, and the specific physiological and molecular mechanisms, are largely unknown.

Chinese jujube (*Ziziphus jujuba* Mill.), native to China, is one of the most important fruit crops in the central and western of China in terms of its economic, ecological, and social importance [22, 23]. The number of jujube cultivars has increased over time, and there are currently more than 1000 documented cultivars in China [24]. Excellent jujube varieties are generally propagated by grafting, and the drought tolerance and saline-alkali tolerance of the trees depends on the rootstock. Sour jujube (Z. *jujuba* var. *spinosa*), a dominant tree in drought and poor environments, is widely used as a rootstock [25, 26]. In previous study, a new germplasm of autotetraploid sour jujube was acquired by colchicine-induced somatic cell chromosomal doubling [27]. Compared with the diploid counterpart, this new germplasm exhibits larger leaves, thicker stems and higher contents of soluble sugar (SS) and soluble protein (SP) [28]. In addition, it had increased salt resistance, owing to the strong regulation of salt-induced osmotic stress and the large positive fold-changes in the expression levels of enriched genes involved in the synthesis and transport of osmotic regulatory substances, and these reactions are also regarded as the main responses to drought [29]. Therefore, we aimed to determine whether the resistance to drought-induced osmotic stress is also enhanced in the autotetraploid and to explore the regulatory mechanisms of genome-wide doubling that underlie its growth advantage under osmotic stress conditions. In this study, we characterized the autotetraploid sour jujube at the phenotypic, physiological and molecular levels. Our findings contribute to the understanding of how autotetraploidization improves plant drought tolerance.

## Results

### Autotetraploid sour jujube shows superior drought tolerance compared with the diploid counterpart

**Fig. 1:**
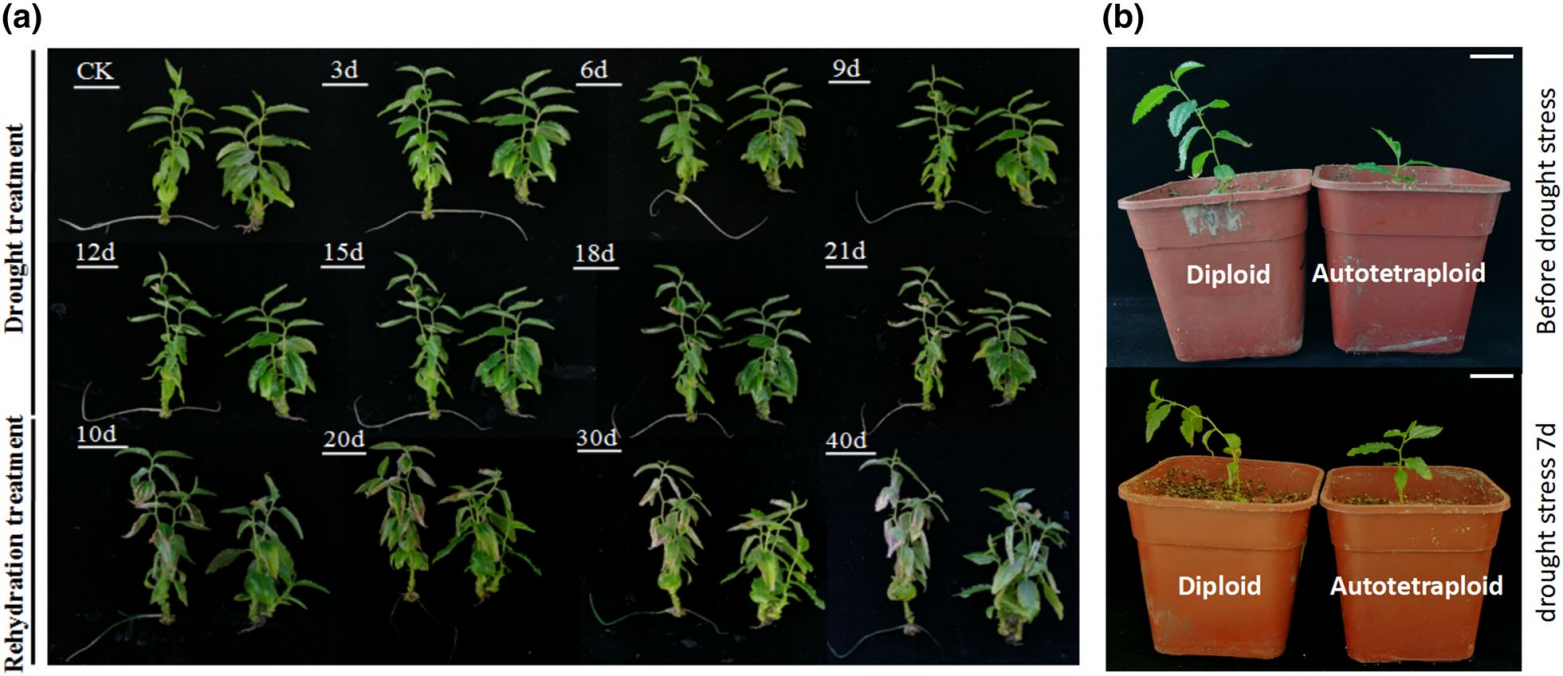
**a** Morphological changes in diploid and autotetraploid sour jujube under drought-stress and rehydration conditions. **b** Phenotypic status of potted diploid and autotetraploid sour jujube plants after 7 days of drought stress. Each image is diploid on the left and the autotetraploid on the right. *Bar* 1.0 cm

To study the drought tolerance of autotetraploid compared with diploid sour jujube, 60 diploids and 60 autotetraploids were subjected to 21 days of drought stress and then 40 days of rehydration treatment. As shown in Fig. 1, the diploid and autotetraploid plants did not change significantly in the first 9 days. From the 12th day, the leaves at the bottom of the diploid plants began to gradually turn yellow, while the leaves of the autotetraploids did not turn yellow until the 21st day. After 21 days, a rehydration treatment was begun to determine whether the two plant types resumed normal growth and to monitor their recovery. However, in the first 20 days after rehydration, more than 80% of the leaves were beginning to turn yellow and wilting on diploid plants, while on the autotetraploids the leaves tips browned but the latter, tillers sprouted from the stem bases. By the 40th day, the main stem growth of more than 40 autotetraploid plants was replaced by the stouter sprouting tillers, while 39 of 60 diploid plants were already dead. In addition, among 30 diploids and 30 autotetraploids growing in drought soil for 7 days, the diploid showed more severely chlorotic leaves and earlier wilting than the autotetraploid. The results indicated that the diploid was more sensitive to drought stress than the autotetraploid and that the autotetraploid had a strong growth restorative ability after drought stress.

**Fig. 2:**
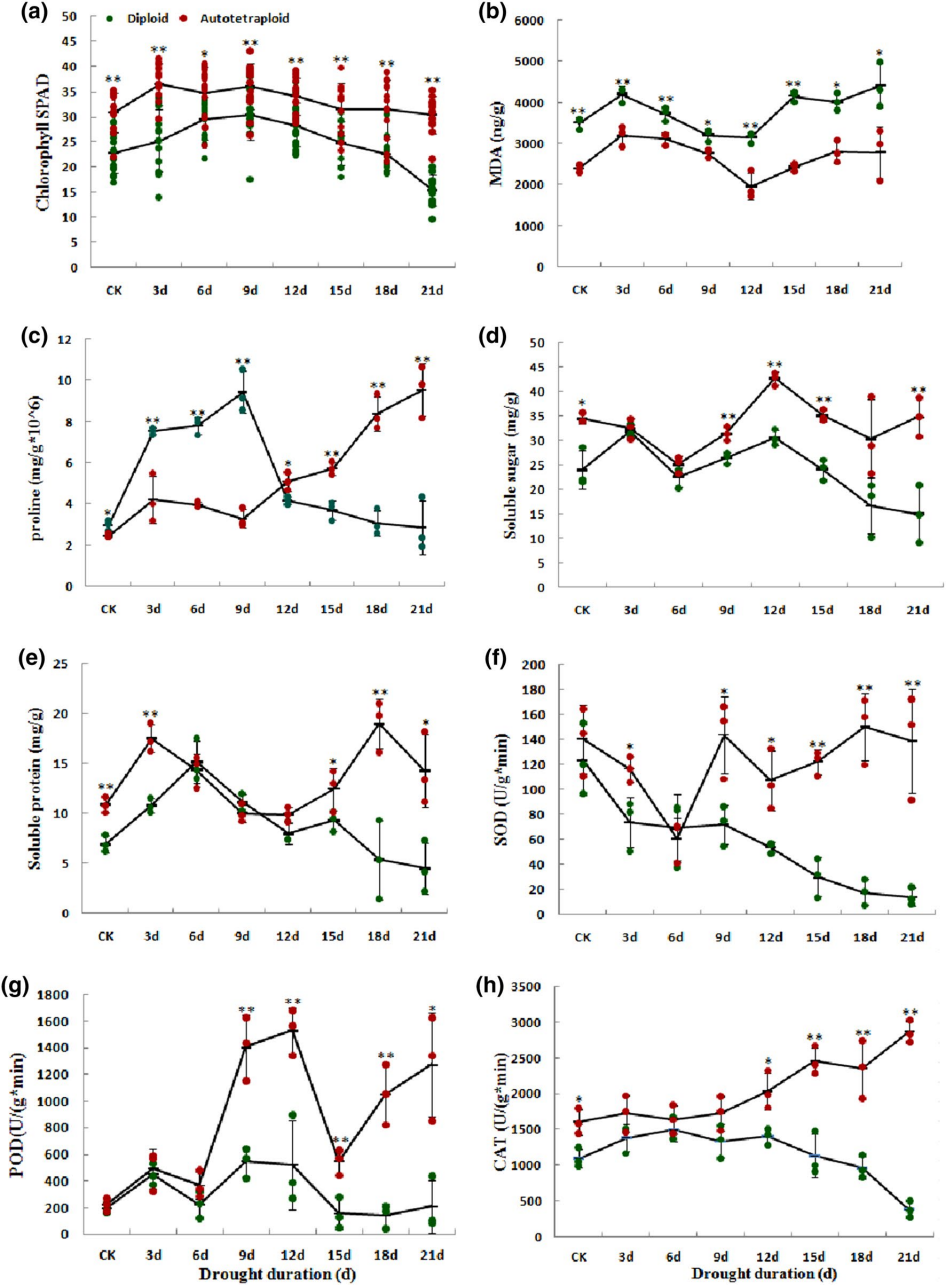
Physiological differences of chlorophyll (**a**), MDA (malonaldehyde) (**b**), proline (**c**), soluble sugar (**d**), soluble protein (**e**), SOD (superoxide dismutase) (**f**), POD (peroxidase) (**g**), and CAT (catalase) (**h**) in diploid and autotetraploid sour jujube plants under control and drought conditions. The dot represents the value of biological repetition. The vertical bars show the standard error. For the difference between diploid and autotetraploid, “*” represents significance level of 0.05 and “**” represents significance level of 0.01

To characterize the difference of physiological responses, eight physiological indicators including the content of chlorophyll, MDA, three osmotic regulators of SS, SP and proline, activity of three antioxidant enzymes of superoxide dismutase (SOD), peroxidase (POD), and catalase (CAT) were measured in diploid and autotetraploid sour jujube under drought-stress conditions (Fig. 2). The leaf tissue samples were collected for determining physiological traits after 0 (untreated, CK), 3, 6, 9, 12, 15, 18 and 21 d of 20% PEG6000 treatment. In the control environment, autotetraploid plants showed a higher chlorophyll, SS and SP contents, as well as CAT activity, compared with diploid plants. On the 3rd day of stress exposure, the SP contents and SOD activity levels in the autotetraploid plants were significantly greater than in the diploid plants. At the beginning of the 9th day of stress, the SS contents, as well as the SOD and POD activity levels in the autotetraploids were significantly greater than in the diploids. On the 12th day of stress, the proline contents and CAT activity levels were also increased. This indicated that mechanisms underlying drought tolerance in diploids and autotetraploids differed, and the resistance level was consistently significant higher in the autotetraploids than in the diploids. In addition, the level of MDA, which reflects peroxidation of membrane lipid, was significantly higher in diploids than in autotetraploids during the whole treatment process.

### Comparison of transcriptional profiles between the autotetraploids and diploids in response to drought stress

To determine the molecular differences underlying drought tolerance in diploid and autotetraploid sour jujube, 24 leaf tissue samples were randomly collected for transcriptome sequencing after 0 (untreated), 6, 12 and 48 h of 20% PEG6000 treatment with diploid material labeled as DCK, D6h, D12h, and D48h and the corresponding the autotetraploid with TCK, T6h, T12h and T48h. A total of 183.99 Gb of clean data was obtained from the diploid and autotetraploid plants, and the Q30 was greater than 93.01%. The statistics of the transcriptome sequencing of all the samples are provided in Additional file 2: Table S1. The transcriptome data were mapped to the ‘Dongzao’ jujube genome, and the efficiency of mapping ranged from 81.87% to 88.73% (Additional file 2: Table S2). Finally, 32,527 genes were obtained, including 3234 new genes. In total, 2546 of the new genes were functionally annotated.

The expression values of all the samples were calculated, and a principal component PCA analysis was conducted to determine correlations between samples (Additional file 1: Figure S1). The aggregation of three biological replicates showed a high correlation among biological replicates. Additionally, the two plant types clustered after 0, 6, 12 and 48 h of drought stress, indicating that homologous polyploidization may lead to smaller inter-gene differential expression levels than stressful environmental treatments.

**Fig. 3:**
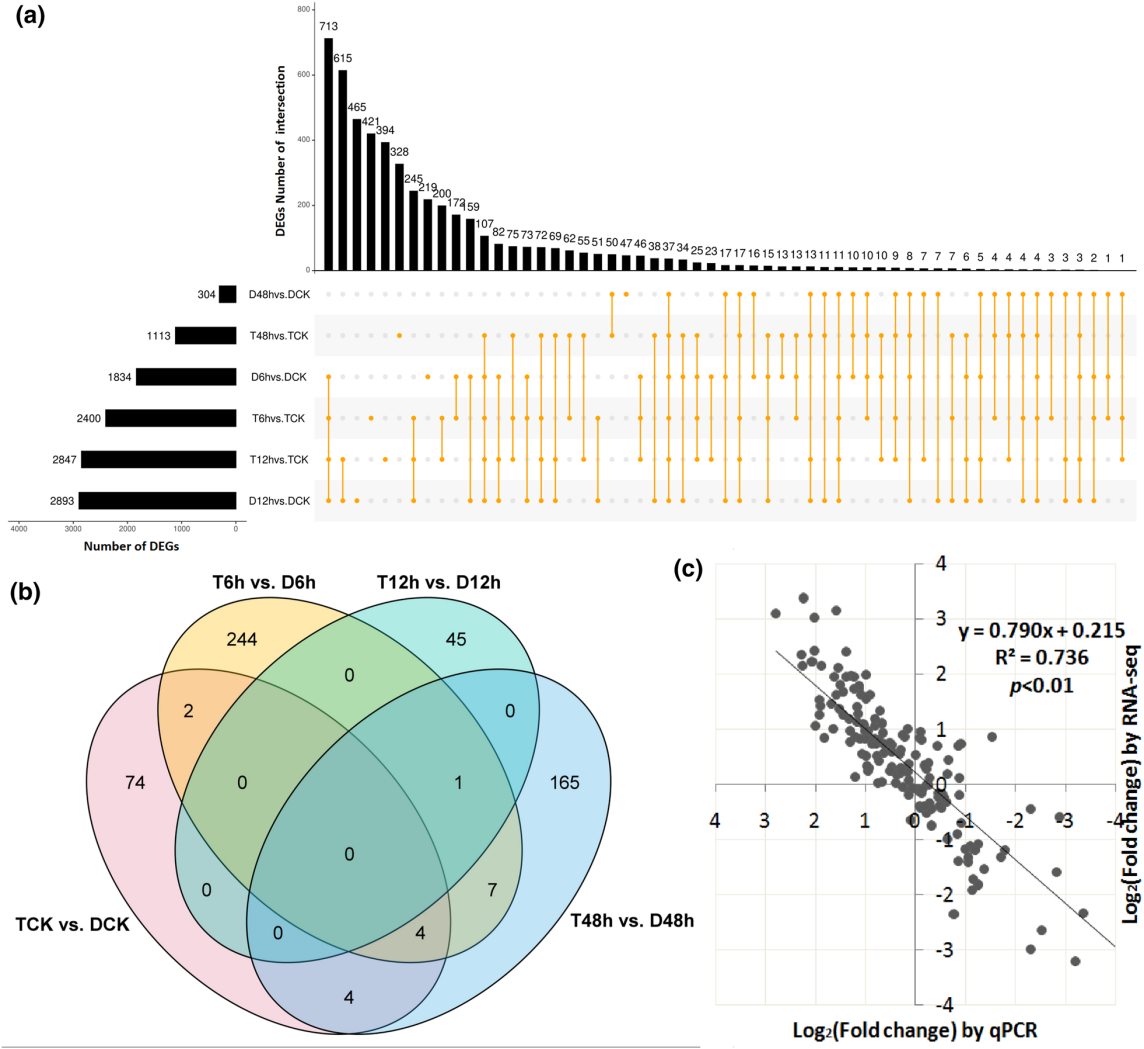
DEGs in diploid and the autotetraploid sour jujube with or without the drought treatment. **a** Upset plot of DEGs in response to drought stress in the diploid and autotetraploid. DCK, D6h, D12h, and D48h represent diploid samples after 0 (untreated), 6, 12 and 48 h of drought treatment and the corresponding samples in the autotetraploid are labeled as TCK, T6h, T12h and T48h. The top column represents the DEGs number of intersections, and the left column graph represents the DEGs number of each dataset. Six separate orange dots represent the DEGs that only present in the one dataset. The DEGs present in at least two datasets is marked as connected orange dots. The connection represents the intersection, and the orange dots on the line represent the corresponding datasets. **b** Statistics of DEGs in diploids and autotetraploids under control conditions and after 6, 12 and 48 h of drought stress. **c** Correlation of expression changes observed by RNA-seq (Y-axis) and qPCR (X-axis)

To investigate DEG responses, the DEGs in the diploid and autotetraploid were determined after exposure to different intensity levels of stress (Additional file 2: Table S3). As shown in Fig. 3a, the DEG numbers in the diploid samples at 6, 12 and 48 h after exposure to drought stress were 1834, 2893 and 304, respectively, while they were 2400, 2847 and 1113, respectively, in the autotetraploid samples. Thus, after 48 h under stress conditions, there was more than three-fold the number of DEGs in the autotetraploid than in the diploid. Overall, there were 1535 specifically expressed genes in the autotetraploid, including 1171, 849 and 929 after 6, 12 and 48 h of stress, respectively, and 930 in the diploid, including 605, 895 and 120 after 6, 12 and 48 h of stress, respectively. Under drought-stress conditions, the numbers of DEGs caused by genome doubling were 258 (T6h vs. D6h), 46 (T12h vs. D12h) and 181 (T48h vs. D48h), of which 178/80, 14/32 and 39/142 were up/down-regulated genes (Fig. 3b, Additional file 2: Table S4). To determine the reliability of the RNA-seq data, genes from different materials were randomly selected for a qRT-PCR analysis. The correlation coefficient between the qPCR and RNA-seq results was high (R^2^ = 0.736, *p* < 0.01), which implied that the expression pattern from the RNA-seq data was reliable and could be further used for the DEG analysis (Fig. 3c).

### Identification of stress-related DEGs from diploid and autotetraploid sour jujube under drought-stress conditions

**Fig. 4:**
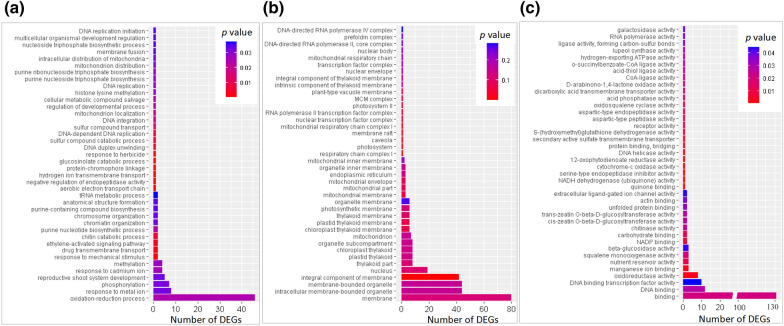
GO annotation in biological process (**a**), cellular component (**b**) and molecular function (**c**) of DEGs from diploid and the autotetraploid under drought treatment

To further reveal the resistance-related functions of DEGs caused by genome doubling, a gene ontology (GO) annotation of D6h vs. T6h, D12h vs. T12h and D48h vs. T48h was conducted. A total of 207, 130 and 211 DEGs were annotated in the GO biological process, cellular component and molecular function categories, respectively. Among the most enriched 40 GO terms (Fig. 4), there were 1 to 46 DEGs involved in pathways of biological process related to stress responses, including ‘oxidation–reduction process’, ‘methylation’, ‘ethylene-activated signaling pathway’, and ‘DNA replication’. In cellular component, there were 1 to 80 DEGs related to ‘membrane’, ‘nucleus’, and ‘photosystem’. In molecular function, there were 1 to 131 DEGs annotated in ‘binding’, ‘DNA binding transcription factor activity’, ‘oxidoreductase activity’, ‘beta-glucosidase activity’ and ‘NADP binding’. These may be the keys to the differences in drought tolerance between diploid and autotetraploid.

### DEGs involved in pathways and altered expression levels in the autotetraploid compared with the diploid under drought-stress conditions

**Fig. 5:**
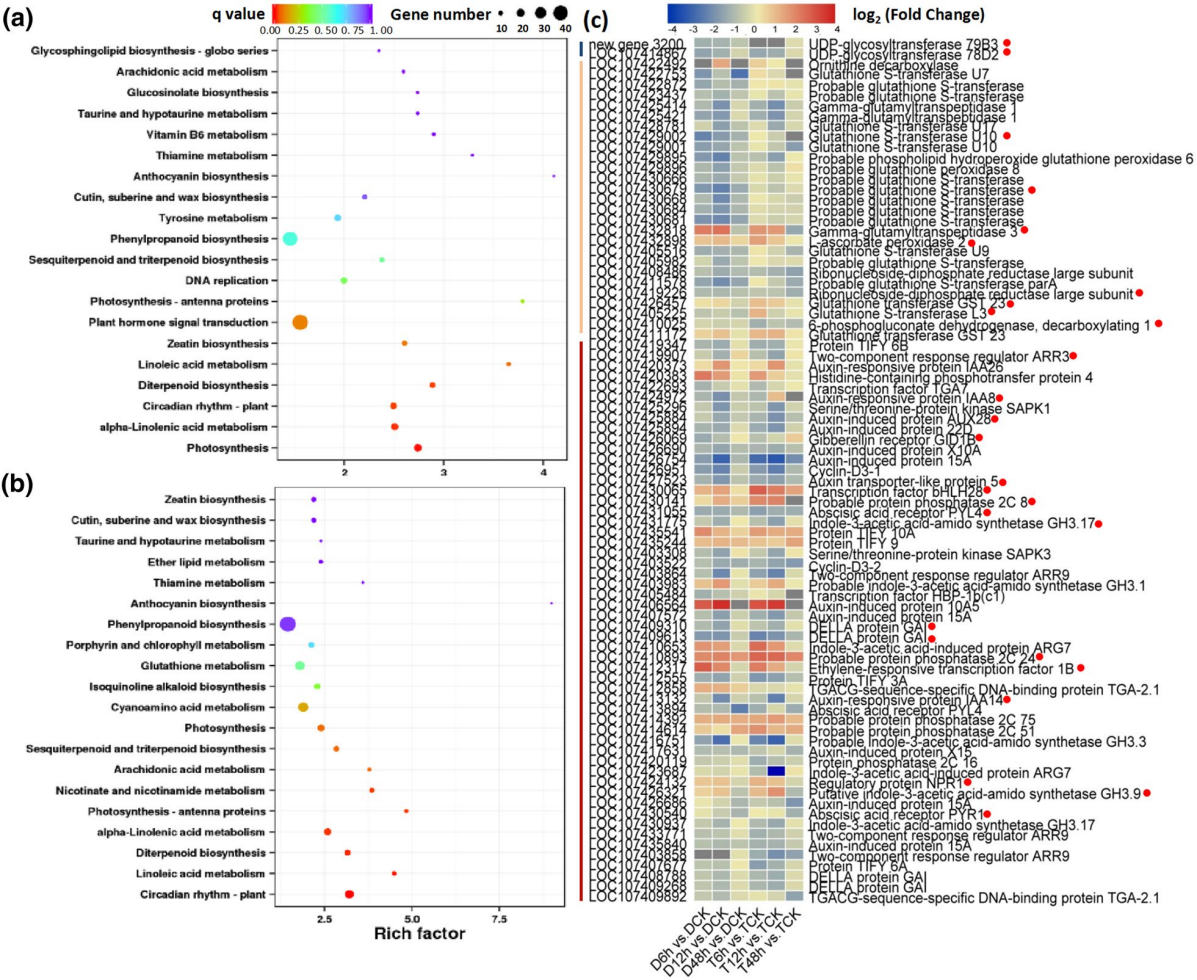
KEGG enrichment of DEGs from autotetraploid (**a**) and diploid (**b**) sour jujube under drought-stress conditions. (**c**) Differential expression levels of DEGs in three pathways. The blue, yellow and red lines on the left represent anthocyanin synthesis, glutathione metabolism and plant hormone signal transduction, respectively. Gray blocks represent the missing value caused by low expression and large repetition difference. Gradient colour bar code at the top indicates log_2_ (Fold change) values with up-regulated genes represented by positive values and down-regulated genes represented by negative values. The red dot after the gene annotation represents the gene mentioned above

To explore the response mechanisms under stress of two ploidy plants, DEGs of the diploid and autotetraploid involved in drought responses were determined and KEGG enrichment of DEGs was performed. The expression level of DEGs involved in stress responses at 6, 12, 48 h after exposure to drought stress compared with the control environment were analyzed. The first 20 pathways with significant enrichment are shown in Fig. 5. In the autotetraploid, most of the DEGs were enriched in anthocyanin biosynthesis, DNA replication, plant hormone signal transduction and photosynthesis (Fig. 5a); however, anthocyanin biosynthesis, glutathione metabolism and photosynthesis were enriched in the diploid (Fig. 5b). Furthermore, the expression levels of 83 genes related to three pathways, anthocyanin synthesis, glutathione metabolism and plant hormone signal transduction, were further analyzed (Fig. 5c, Additional file 2: Table S5). After the drought treatment, two anthocyanin-related genes encoding UDP-glycosyltransferase (New gene 3200 and *LOC107414867*) were down-regulated in both the diploid and autotetraploid. In total, 19 of the 27 glutathione metabolism-related DEGs, including glutathione S-transferase genes (*LOC107429002* and *LOC107430679*), were down-regulated in the diploid, while only 9 genes, including ribonucleoside-diphosphate reductase large subunit (*LOC107419226*) and 6-phosphogluconate dehydrogenase (*LOC107410025*), were down-regulated in the autotetraploid. Genes encoding gamma-glutamyltranspeptidase 3 (*LOC107432818*) and l-ascorbate peroxidase 2 (*LOC107432898*) were up-regulated in both plant types; however, the genes encoding glutathione transferase GST 23 (*LOC107426457*) and glutathione *S*-transferase L3 (*LOC107405225*) were up-regulated 2.0- and 2.4-fold, respectively, only in the autotetraploid. DEGs related to plant hormone signaling, such as the Aux-related genes encoding two-component response regulator ARR3 (*LOC107419907*), Aux-induced protein AUX28 (*LOC107425884*), Aux transporter-like protein 5 (*LOC107427523*), indole-3-acetic acid-amido synthetase GH3.17 (*LOC107431775*) and Aux-responsive protein IAA14 (*LOC107413132*), were down-regulated in plants of both ploidy levels after exposure to drought stress. However, the gene encoding Aux-responsive protein IAA8 (*LOC107424972*) was down-regulated in the diploid, but up-regulated in the autotetraploid. The genes encoding the regulatory protein NPR1 (*LOC107424132*) and putative indole-3-acetic acids-amido synthetase GH3.9 (*LOC107426321*) showed no differences in the diploid, but were up-regulated in the autotetraploid. The gene encoding gibberellin (GA) receptor GID1B (*LOC107426069*) was down-regulated in the diploid, but showed no differential expression in the autotetraploid. Two of four DEGs encoding DELLA protein GAI (*LOC107409310* and *LOC107409613*) were down-regulated in at both ploidy levels, while the other two were only down-regulated in the autotetraploid. The SA-related gene *NPR1* (*LOC107424132*) was up-regulated in the autotetraploid but showed no differential expression in the diploid after stress exposure. The expression levels of ethylene-regulated genes were also altered, such as the gene encoding ethylene-responsive TF 1B (*LOC107412317*), which was up-regulated in both the diploid and autotetraploid. The JA response-related DEG encoding protein TIFY 3A (*LOC107412555*) was down-regulated in both the diploid and autotetraploid, and the bHLH28 (*LOC107430065*) was up-regulated in T6h vs. TCK, T12h vs. TCK, T48h vs. TCK, D6h vs. DCK and D12h vs. DCK, but not in D48h vs. DCK. Moreover, ABA-related DEGs encoding protein phosphatase 2C 8 (*LOC107430141*) and protein phosphatase 2C 24 (*LOC107410893*) were up-regulated, while the gene encoding ABA receptor PYL4 (*LOC107431055*) was down-regulated in both two materials. Nevertheless, ABA receptor PYR1 (*LOC107430540*) was down-regulated in the autotetraploid but showed no differential expression in the diploid. Eight genes encoding UGT78 (*LOC107414867*), L-ascorbate peroxidase 2 (*LOC107432898*), IAA8 (*LOC107424972*), bHLH28 (*LOC107430065*), PP2C 51 (*LOC107414614*), NPR1 (*LOC107424132*), GH3.9 (*LOC107426321*) and DELLA (*LOC107408788*) were selected to further validate their expression levels during drought treatment using a qRT-PCR analysis (Additional file 1: Figure S2). There were similar expression trends for these genes in the diploid and autotetraploid after exposure to drought stress. However, there were considerable differences between the two ploidy materials, including the expression levels of *LOC107424972* at 12 and 48 h, *LOC107430065* at 6, 12 and 48 h and *LOC107408788* at 12 h of drought treatment. These results suggested that there were many similarities and varied differences in responses to stress between the diploid and its corresponding autotetraploid. These pathways and their corresponding DEGs might be key factors that lead to the difference in drought tolerance between the autotetraploid and diploid.

### ROS scavenging enzyme-related DEGs were specifically expressed in autotetraploid sour jujube under drought-stress conditions

**Fig. 6:**
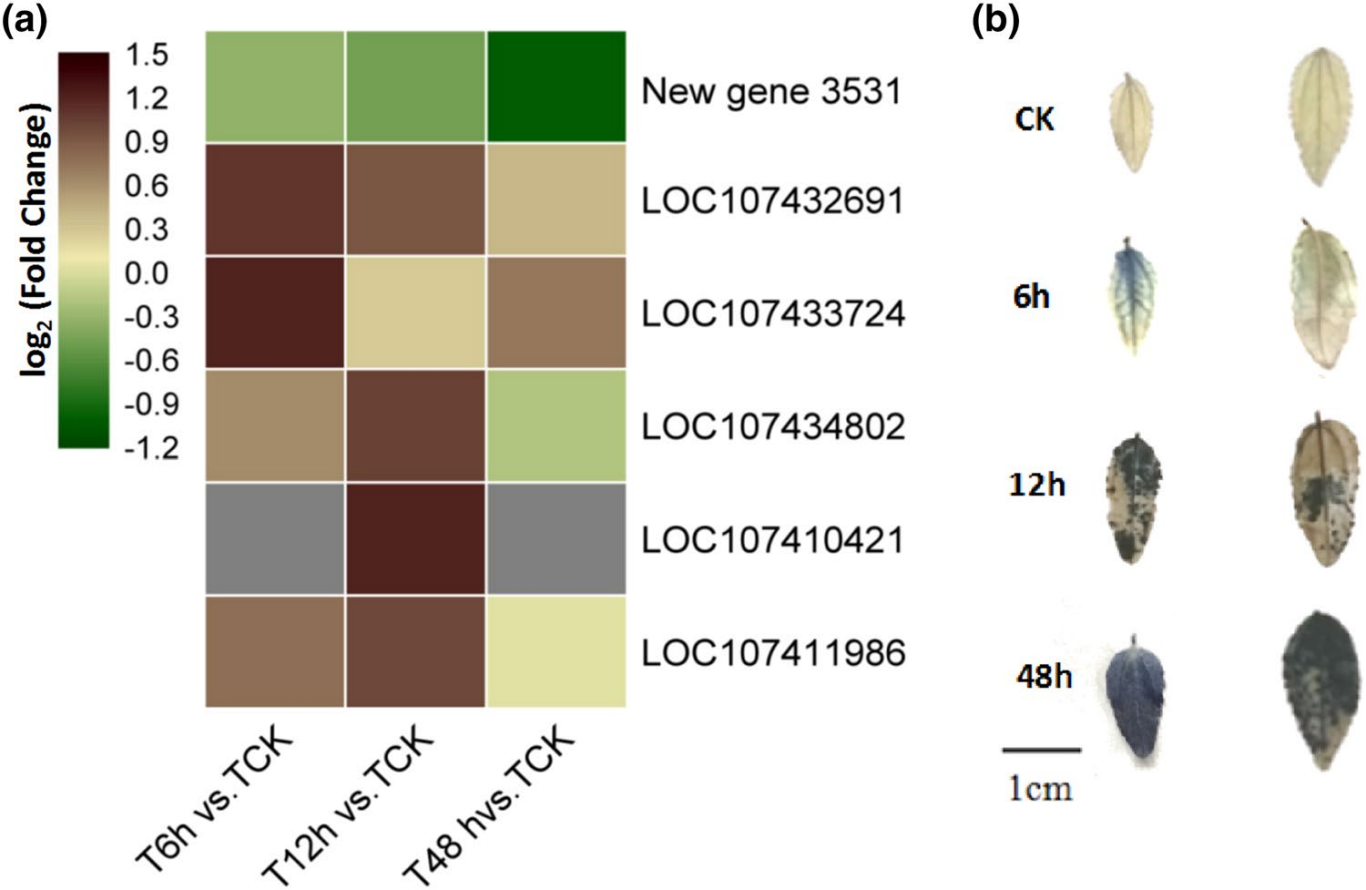
Analysis of ROS scavenging-related DEGs in diploid and autotetraploid sour jujube (**a**). Specific DEGs related to ROS scavenging in the autotetraploid. Gray blocks represent the missing value caused by low expression and large repetition difference. Gradient colour bar code at the left indicates log_2_ (Fold change) values with up-regulated genes represented by positive values and down-regulated genes represented by negative values. **b** Diploid (left) leaf produced a deeper staining color than autotetraploid (right) leaf during the drought treatment. The deeper staining of leaves represents a higher cellular peroxidate content

In total, 12 and 15 DEGs in the diploid and autotetraploid sour jujube involved in peroxisome function were obtained. To further explore the regulatory mechanisms of the antioxidant system that enhanced autotetraploid stress resistance, the expression differences of nine DEGs common to both the diploid and autotetraploid (Additional file 2: Table S6) and six DEGs specific to the autotetraploid were analyzed (Fig. 6). Most of the common genes had similar differential expression fold changes. There were six DEGs encoding antioxidant enzymes, such as *SOD* (*LOC107432691*) and *LACS7* (*LOC107411986*), that specifically existed in the autotetraploid, and five were up-regulated under drought conditions. The expression levels of these six specific genes were further verified by qRT-PCR to determine candidate genes (Additional file 1: Figure S3). These genes were characterized by high-fold differential expression-level changes. In particular, *LOC107434802* and *LOC107411986* in the autotetraploid were expressed at significantly higher levels than that in the diploid when exposed to the same intensity of drought stress. These differences might be keys to the superior ROS scavenging ability in the autotetraploid compared with the diploid. Correspondingly, the cellular peroxidate contents in the diploid and autotetraploid leaves after stress were also visualized by tissue staining (Fig. 6b). The diploid leaves were almost filled with peroxidate after 6 h of the drought stress, while the peroxidate staining in the autotetraploid appeared after 12 h of drought stress. The whole leaves sampled from the same position on both diploid and the autotetraploid plants were stained after 48 h of stress but the diploid leaves were a darker blue than the autotetraploid leaves.

### Enriched DEGs encoding TFs in diploid and the autotetraploid sour jujube during drought stress

**Fig. 7:**
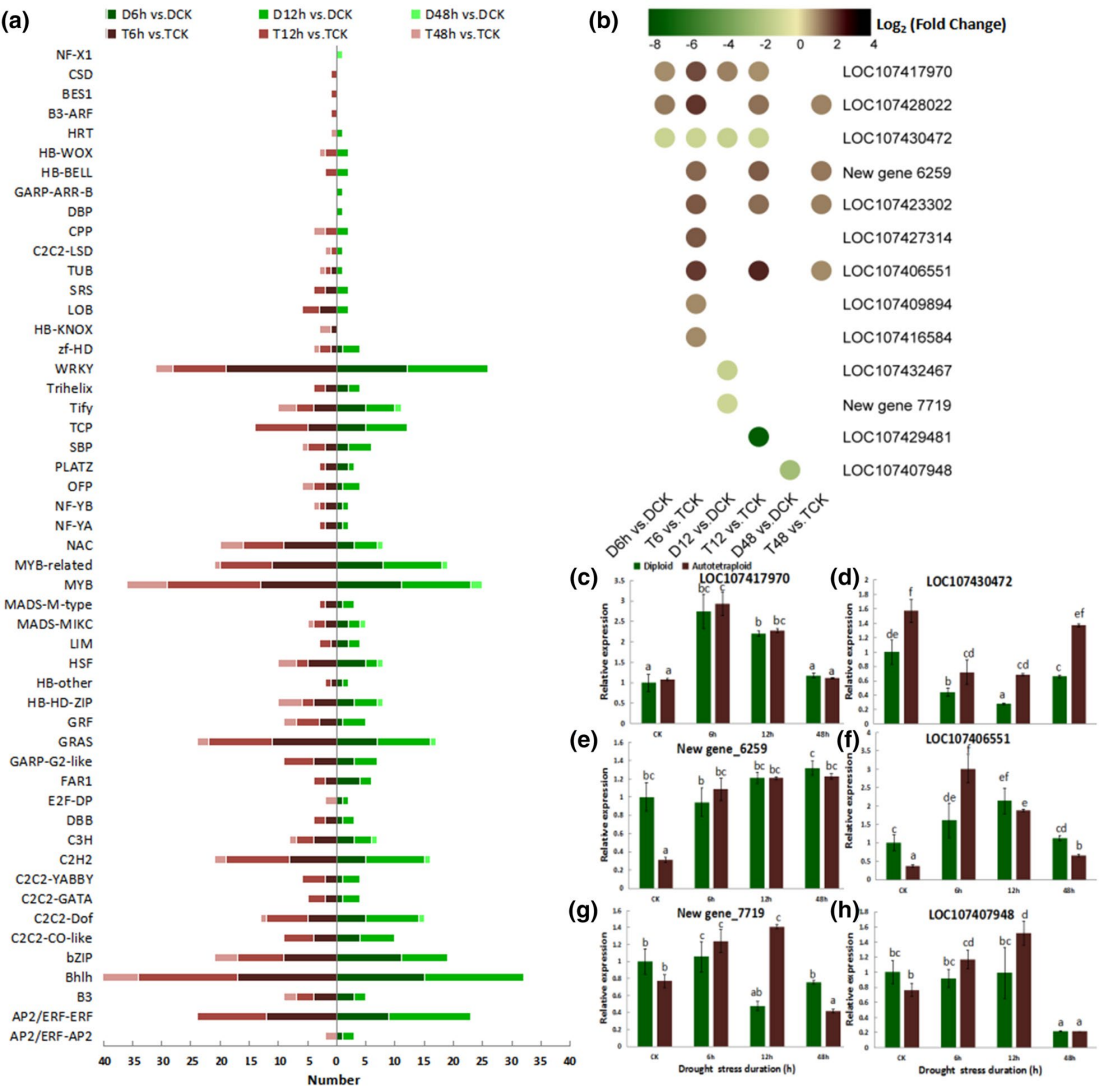
Analysis of DEGs encoding TFs in diploid and autotetraploid sour jujube. **a** The numbers of TF genes differentially expressed in the diploid and autotetraploid at 6, 12 and 48 h after drought treatment. **b** The differential expression levels of *NAC* genes in the diploid and autotetraploid under drought-stress conditions. Gradient colour bar code at the top indicates log_2_ (Fold change) values with up-regulated genes represented by positive values and down-regulated genes represented by negative values. **c**–**h** Verification of differential expression levels of six *NAC* genes *LOC10747970* (**c**), *LOC107430472* (**d**), New gene 6259 (**e**), *LOC107406551* (**f**), New gene 7719 (**g**) and *LOC107407948* (**h**). The vertical bars show the standard error and a significance level of 0.05 was used for different letters above bars

To explore the roles of TFs on drought response, DEGs encoding TFs in the diploid and autotetraploid were predicted (Fig. 7a). A total of 1,686 TFs were identified. In general, the numbers of DEGs encoding WRKY, NAC, MYB, MYB-related, GRAS, C2H2, bZIP, Bh1h and ERF TFs in the diploid and autotetraploid were determined, and more of these DEGs were found in the latter than in the former. Notably, after 6 h of drought stress, there were 11 and 7 DEGs encoding GRAS, 9 and 3 *NAC*, and 19 and 12 *WRKY* genes were identified in the autotetraploid and diploid, respectively. After 12 h under stress conditions, 16 and 12 *MYB*, 7 and 4 *NAC*, and 9 and 14 *WRKY* genes were identified in the autotetraploid and diploid, respectively. After 48 h of drought stress, there were only 12 types of TF DEGs in the diploid, but 29 types in the autotetraploid, and 7 *Bhlh* genes were found in the autotetraploid but not in the diploid. Seven and four genes encoding MYB and NAC were differentially expressed in the autotetraploid but only two and one were differentially expressed in the diploid. The numbers of *NAC* TF genes were different at 6, 12 and 48 h after stress exposure in the two plant types. Therefore, their expression level changes were analyzed (Fig. 7b). In total, 9 of 13 genes were differentially expressed in T6h vs. TCK, and eight were up-regulated 2.2- to 4.2-fold. Only one diploid and four autotetraploid *NAC* genes showed differential expression changes with 2.1- and 2.4-fold after 48 h of drought stress. In addition, *LOC107429481* was down-regulated almost 150-fold after 12 h of drought stress in the autotetraploid, but no significant difference was found in the other comparison groups. Finally, the qRT-PCR analysis of the six *NAC* genes was performed (Fig. 7c-h). *LOC107430472*, *LOC107406551*, New gene 7719, and *LOC107407948* were differential expressed in the diploid and autotetraploid after exposure to drought stress.

### Hub genes involved in the drought-resistance difference between diploid and autotetraploid sour jujube

**Fig. 8:**
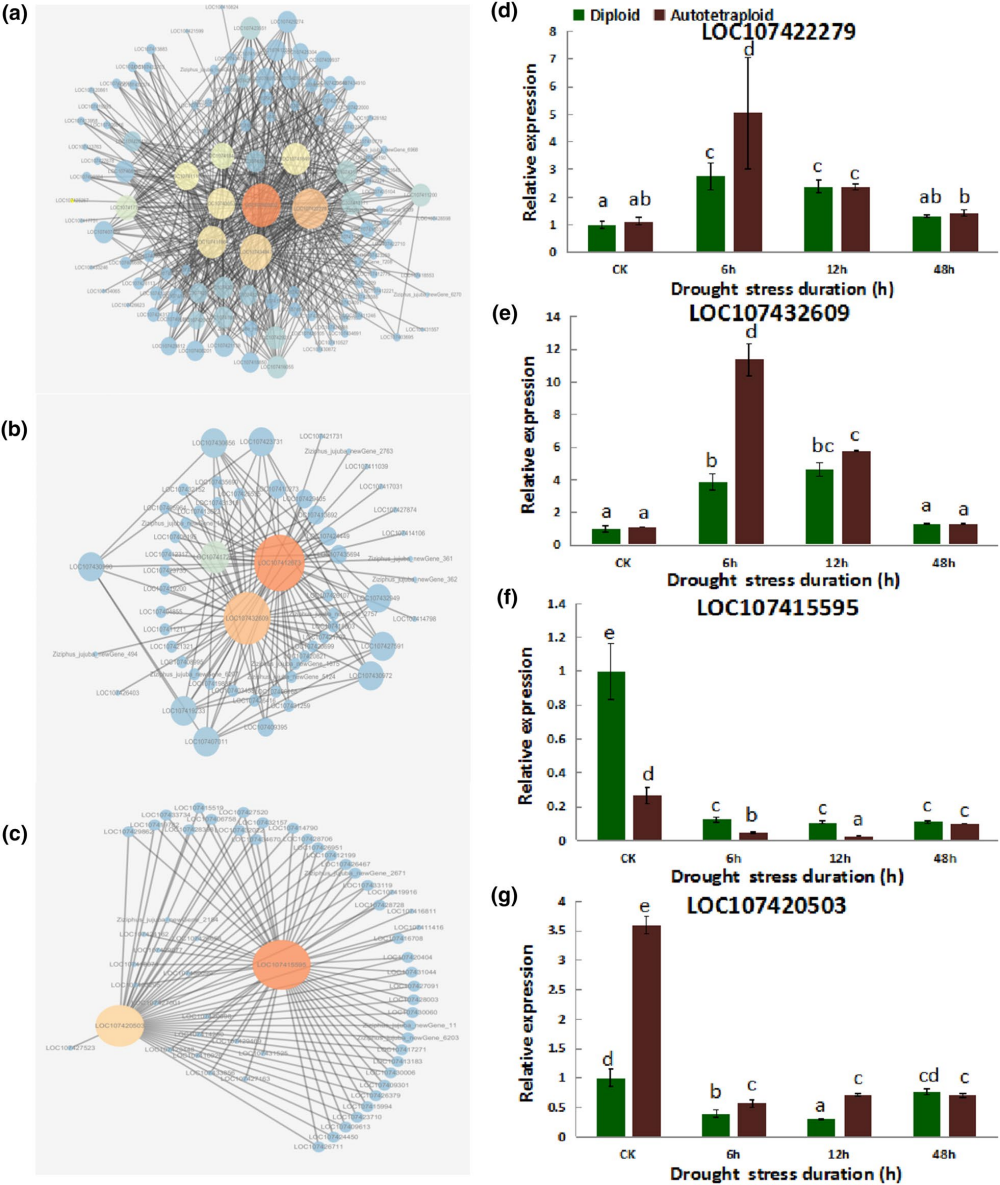
Gene co-expression network and hub gene expression analysis. Gene co-expression network of the positively correlated turquoise (**a**) and green (**b**) modules and of the negatively correlated blue module (**c**). The size of the circle represents the number of linked genes (i.e., a bigger circle has more linked genes); the circle color represents the gene connectivity, and orange and blue represent the strongest and weakest connectivity, respectively; the lines between circles indicate correlations between genes. (**d**–**g**) Gene expression levels from qRT-PCR for the four hub genes *LOC107422279* (**d**), *LOC107432609* (**e**), *LOC107415595* (**f**) and *LOC107420503* (**g**). The vertical bars show the standard error and a significance level of 0.05 was used for different letters above bars

The central control network for diploid and autotetraploid differences in response to low water potential and the hub genes that affect the superior drought tolerance in the autotetraploid were investigated by a weighted gene co-expression network analysis (WGCNA). As shown in Additional file 2: Table S7, 18 TF genes with higher differential expression level in the autotetraploid compared with in the diploid with and without drought treatment were selected as bait. All the DEGs of the diploid and autotetraploid before and after exposure to stress formed a pool were listed in Additional file 2: Table S8. A total of seven modules were found and the genes with kME > 0.7 were selected as the members in each module (Additional file 2: Table S9). After comparing the correlation between modules and baits, the three most relevant modules, turquoise, green and blue were selected to analyze (Additional file 1: Figure S4). The genes encoding the NAC, LOB, MYB and WRKY TFs were positively correlated with the turquoise and green modules. The co-expression network was constructed for turquoise (weight > 0.59, Fig. 8a) and green (weight > 0.45, Fig. 8b) modules. The hub genes with strongest connectivity that located in the regulatory network center were determined, as follows: *LOC107403632* (glycine-rich RNA-binding protein), *LOC107422279* (glycine-rich RNA-binding protein 2) and *LOC107434947* (ABC transporter G family member 34) in turquoise module, *LOC107412673* (G-type lectin S-receptor-like serine/threonine-protein kinase RLK1) and *LOC107432609* (B-box zinc finger protein 22) in green module. The negatively correlated module for the DEGs encoding NAC, LOB, MYB, WRKY and FAR1 TFs was blue, and the gene co-expression network was also constructed (weight > 0.49, Fig. 8c). The hub genes were identified as *LOC107420503* and *LOC107415595*, which both encode uncharacterized protein. Furthermore, qRT-PCR was performed for four hub genes. These genes were differentially expressed between the diploid and autotetraploid after 6 h and 12 h of drought treatment. Interestingly, these two negatively correlated hub genes, *LOC107415595* and *LOC107420503* under control conditions, were also unexpectedly differentially expressed in two ploidy plants.

## Discussion

### Autotetraploid jujube exhibited superior drought tolerance and regrowth potential under drought stress

Gene duplication events, especially whole-genome duplications, quickly create redundant modules in the genome and provide the possibility for genotypic and phenotypic changes [30]. Moreover, the increase in genetic diversity and the buffering effect of gene redundancy make polyploids or polyploid populations more adaptive to extreme environments [16]. In this study, the autotetraploid sour jujube showed a superior drought tolerance compared with its diploid counterpart. Many phenotypes are interlinked and many observed changes may represent pleiotropic effects caused by the underlying disturbances. The significant up-regulation of genes in autotetraploids have been reported to be involved in auxin signaling [28]. Here, the drought treatment inhibited autotetraploid growth but rehydration renewed sprouting tiller growth, and this may also be mediated by regulating auxin signaling. Additionally, polyploids are generally characterized by slow growth and dwarfism, resulting in lower resource utilization, and these characteristics may result in polyploids suffering less cell damage [31]. In our study, the significantly lower MDA content suggested less oxidative damage to cell membranes in the autotetraploid compared with the diploid during the drought treatment. Moreover, stomatal size and density may account for differences in the transpiration rate, drought tolerance and water-use efficiency [32–36]. Polyploid plants, including the autotetraploid sour jujube, usually have large low-density stomata [37, 38]. In autotetraploids *Arabidopsis*, the reduced transpiration rate mediated by enhanced stomatal closure and the reduction in the stomatal index is related to increased drought tolerance. In addition, the changes in the stomatal characteristics increase the gas exchange rate, and the number of chloroplasts, as well as the photosynthetic rate, in each cell increases in the enlarged leaves. In the autotetraploid sour jujube, a high chlorophyll content was detected, and DEGs were enriched in the photosynthetic pathway. Thus, polyploidization may improve a plant’s ability to adapt by adjusting the biofilm system, as well as the size and structure of its cells, to reduce photosynthetic damage. Therefore, polyploids, with their increased osmotic stress resistance, may withstand a variety of environmental stresses that induce osmotic stress in cells, suggesting that the autotetraploid sour jujube may have great potential as a rootstock for Chinese jujube, which warrant further investigation.

### Whole-genome duplication mediated greater transcriptome changes under drought stress in jujube

Plant polyploidy improves adaptability by adjusting cellular and organismal homeostasis and cellular processes to a new growth state [39]. Compared with in diploids, abiotic stress leads to greater transcriptome changes in polyploids [40]. In sour jujube, there were large difference from the number and function of DEG in diploid and autotetraploid under drought stress. These differences may be a result of gene dose effects in which have an increased DEGs. Another possibility is gene-dose compensation effect induced by the divergence in DNA methylation [41], microRNAs [42], histone modifications [15] and/or alternative splicing patterns [43] after a whole-genome duplication. Thus, the gene dose and gene-dose compensation effects in autopolyploid sour jujube genomes may be mechanisms of transcriptome changes and they may play important roles in the drought tolerance of the autotetraploid sour jujube.

### Whole-genome duplication mediated osmotic regulators accumulation under drought stress in jujube

Whole-genome duplication events lead to multi-gene biological networks, which may establish new metabolic, regulatory or developmental pathways that enhance plant adaptation. Osmotic stress induces plants to accumulate a variety of active osmotic compounds, such as proline and sugar, to enhance their ability to perform osmotic adjustments [44, 45]. In our study, during drought stress, there were significantly high soluble sugar, soluble protein and proline contents, while abundant drought-resistant genes were involved in the synthesis and transport of osmotic regulatory substances, such as phenylpropanoid, sugar, proline and anthocyanin, which may lead to the autotetraploid’s superior resistance to osmotic stress compared with the diploid. Anthocyanin plays an important role in increasing drought resistance [46]. The down-regulation of *UGT78*, which may be involved in the synthesis and transport of anthocyanin in vacuoles [47], was greater in the diploid than in the autotetraploid under stress conditions, indicating that it may mediate the decreased synthesis of anthocyanin. Thus, one reason for the enhanced osmotic regulatory capacity after genome-wide doubling is the extensive accumulation of osmotic regulators, which has been reported by Wei et al. [48] and Zhu et al. [49].

### Whole-genome duplication mediated regulation and crosstalk of hormones under drought stress in jujube

Plant hormones are involved in a variety of stress responses, and applying hormones with specific functions may significantly alter plant characteristics [50, 51]. For example, compared with their diploid counterparts, ABA metabolism-related and endogenous cytokinin-, GA-, ethylene- and Aux-related genes in tetraploids of *Lycium ruthenicum*,* Citrus limonia* and mulberry are significantly differentially expressed and result in enhanced resistance and strong growth [52–54]. In this study, the DEGs related to five types of hormones, i.e. Aux, ABA, GA, SA and JA, showed different responses in the two ploidy-level sour jujube, indicating that plant hormones may play key roles in the improved drought tolerance of the autotetraploid. In the autotetraploid, up-regulated *IAA8* and *GH3.9* might regulate signaling and accumulation of Aux to promote tiller growth, and mediate abiotic stress resistance [55, 56]. DELLA proteins related to GA signaling are key factors in stress responses, including perceiving and responding to water deficits [57–59], ROS scavenging [60] and mediating hormonal cross-talk between GA- and ABA-signaling pathways [61]. Interestingly, in our study, *PP2C 51* in ABA-signaling, and *LOC107408788* and *LOC107409268*, encoding the DELLA protein GAI in the GA-signaling pathway, were only differentially expressed in the autotetraploid under stress conditions. These results suggest that hormones may play an important role in the change of drought tolerance including sensing, conducting and regulating cross-talk after the whole-genome doubling.

### Whole-genome duplication regulated the adaption of jujube to oxidative stress under drought stress

Environmental stress interferes with the normal metabolism of cells, disturbs the balance between the production and elimination of ROS, and induces ROS accumulation, resulting in oxidative stress [62]. Polyploids may have low levels of cellular stress and higher abilities to scavenge ROS and produce antioxidants, and these characteristics may result in their general adaptability to stress [31, 63–65]. During drought stress, some DEGs involved in oxidation–reduction process were detected in sour jujube and its autotetraploid. Here, the genes encoding glutathione transferase and ascorbate peroxidase in glutathione metabolism pathways showed larger positive fold-changes of the expression levels in the autotetraploid than in the diploid, indicating that they were involved in the enhanced drought tolerance [5, 66]. Additionally, *SOD* and *LACS7* were specifically up-regulated in autotetraploid plants, in which *SOD* may have reduced the peroxide level and mediated oxidative stress tolerance [67, 68], while *LACS7* may have induced drought tolerance by promoting plant growth [69]. Furthermore, the significantly higher antioxidant enzyme activities of POD, SOD and CAT, as well as the lower ROS level, in the autotetraploid under stress conditions, indicating that a higher level of enzymatic responses in ROS scavenging may be induced by osmotic stress in autotetraploid. These results indicate that the neo-functionalization of multi-copy genes after whole-genome doubling might have established an extra stress response mechanism in the autopolyploid and activated the enzymatic system, thereby further increasing the polyploid’s ability to adapt to osmotic stress [70, 71].

### Transcription factor regulation networks improved drought tolerance of autotetraploid jujube

After a whole-genome duplication event, the expansion of specific gene families and the resulting dose effects, particularly the biased retention of genes with regulatory and developmental functions, may promote plant adaptation to the environment [72, 73]. As transcriptional regulators, TFs promote or inhibit gene expression through targeted binding functions, thereby affecting the synthesis, transport and metabolism of functional proteins [74, 75]. Generally, the presence of more differentially expressed genes and a complicated regulatory network as results of chromosomal doubling might allow multi-strategy responses to stress in the autotetraploid [76]. Multiple TFs such as NAC, WRKY, bZIP and MYB are involved in the difference of stress response between diploid and tetraploid [77, 78]. In this study, the total number of differential TF genes, including WRKY, NAC, MYB, GRAS, C2H2, bZIP, Bh1h and ERF, in the autotetraploid was more than in the diploid, which may be the reason of the resistance difference. Plant-specific NAC proteins regulate cell division and multiple biological processes in plants, including secondary wall formation [79], leaf senescence [80], and stress responses [81]. In the present study, New gene 6259 and *LOC107406551*, which is homologous to *ANAC072*, were up-regulated in the autotetraploid during the stress treatment, may response to drought treatment through binding to a drought-responsive *cis*-element [82]. New gene 7719, homologous to *SUPPRESSOR OF GAMMA RESPONSE 1*, showed significant difference between the two plant types, which may enhance resistance in the autotetraploid through a signaling cascade that response to ROS-induced DNA damage [83]. In addition, the differential expression of genes (*LOC107430472* and *LOC107407948*) involved in secondary wall formation that occurred in the diploid and autotetraploid during drought stress was similar to that of *Betula platyphylla*, which presented enhanced salt and osmotic stress tolerance levels in *BpNAC012*-overexpression transgenic birch lines [84]. Thus, multiple and increasingly differentially expressed *NAC* genes may regulate enriched pathways for osmotic stress tolerance in the autotetraploid. Furthermore, whole-genome duplication events lead to the co-development of genes (e.g. interaction or co-expression), especially in global genes regulatory networks that are formed by highly connected genes that are co-expressed and/or co-regulated by the same set of key regulators [85]. In *Arabidopsis*, glycine-rich RNA-binding protein 2, containing oxidative stress-induced RNA-binding sites [86], affected seed germination under salt-stress conditions, but did not show influence under osmotic stress conditions [87]. However, this gene was found induced by drought-stress conditions and was identified as hub gene in our study that may be a result of the doubling homologous chromosomes. Moreover, B-box zinc finger protein 22 in *Arabidopsis* regulated the expression of genes responsive to light hormone signals, contributing to optimal seedling development [88] and de-etiolation [89]. Furthermore, it was shown to participate in cross-talk with multiple hormonal signaling pathways, including Aux, GA_3_ and ABA [90–92]. Thus, the gene encoding B-box zinc finger protein 22 may promote the development of autotetraploid plants to sustain vegetation in response to stress, and be involved in the regulation of multiple hormone cross-talk and transport-related pathways. This could further prove the genome-wide doubling leading to cross-talk of multi-hormone during drought tolerance. However, further functional verification is still required.

## Conclusion

**Fig. 9:**
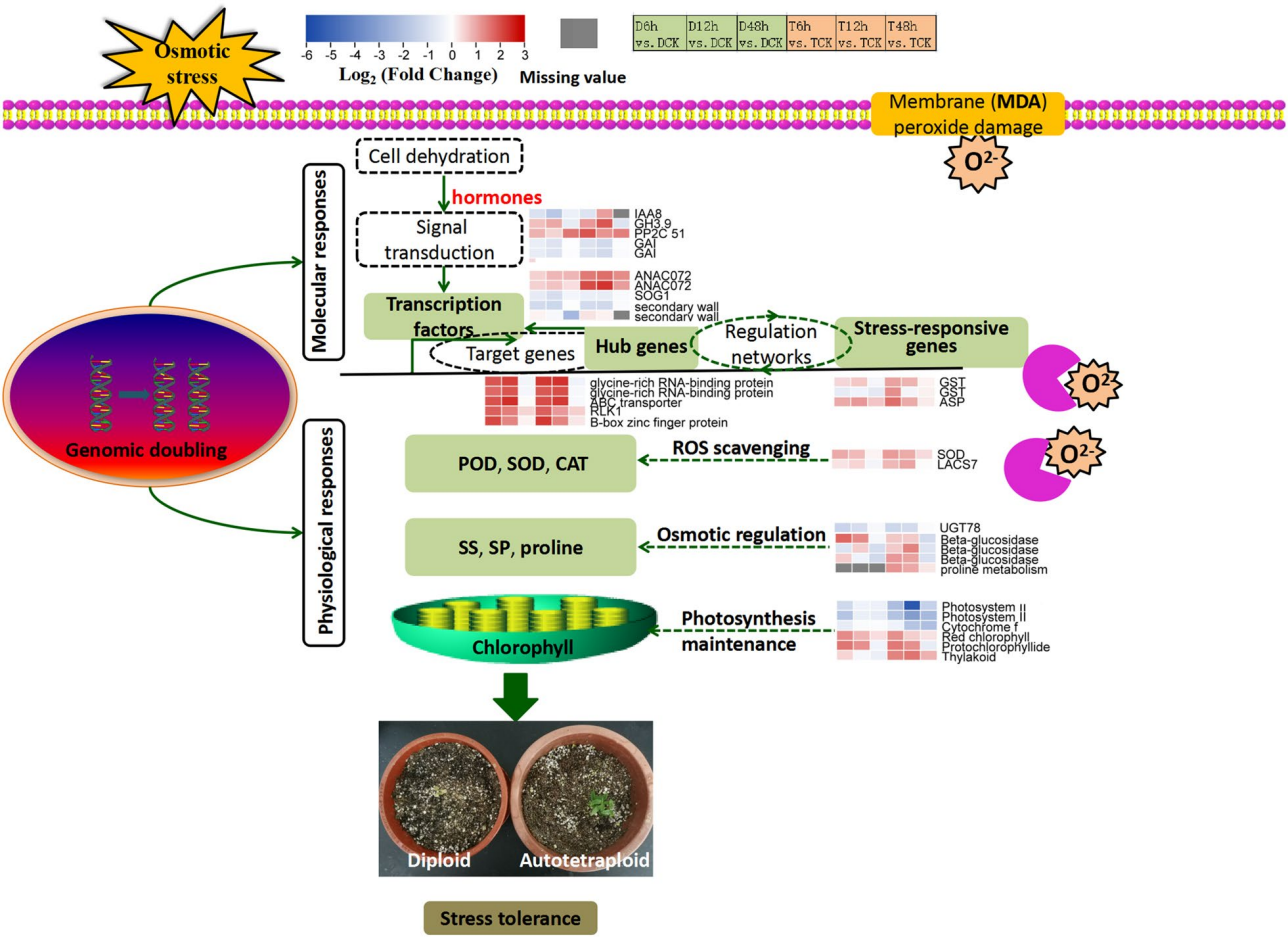
Putative model of the internal mechanisms of superior tolerance after chromosomal doubling. Gray blocks represent the missing value caused by low expression and large repetition difference. Each heatmap represents the differential expression level based on log_2_ (Fold change) value. Gradient colour bar code at the top indicates log_2_ (Fold change) values with up-regulated genes represented by positive values and down-regulated genes represented by negative values

In conclusion, this study revealed that the autotetraploid *Z. jujuba* Mill. var. *spinosa* exhibited enhanced drought tolerance compared to its diploid counterpart. The change in drought-resistance pathways resulting from whole-genome doubling were preliminarily revealed and summarized (Fig. 9). During drought stress, water deficit signals are received by cells, further stimulate hormonal responses, including those of Aux, ABA, SA and GA, and active downstream signaling enzymes. Afterwards, TFs, including NAC, WRKY, bZIP and MYB are induced to regulate the transcription of target genes. Several hub genes (i.e., genes encoding Glycine-rich RNA-binding protein 2 and B-box zinc finger protein 22 and *LOC107415595*, *LOC107420503*) may be regulated by or in turn regulate TFs. Finally, the proteins encoded by the stress-response genes, such as *UGT78*, *GST*, *SOD* and *LACS7* may complete their specific functions, affecting the physiological responses, including changing the capability of osmotic regulation, the ROS scavenging and photosynthesis maintenance. Owing to the whole-genome doubling, many functional genes in the autotetraploid plants were differentially expressed compared with in the diploid during drought stress, resulting in resistance difference. The ultimate origin of the large differences in hormones, osmotic regulators, antioxidant systems and photosynthesis-related pathways between diploid and autotetraploid sour jujube will be further investigated.

## Methods

### Plant materials and stress treatment

The autotetraploid sour jujube in this study was obtained by Cui et al. [27] and was preserved in National Engineering Laboratory for Tree Breeding, Beijing Forestry University, Beijing, China. All seedlings used in this experiment were grown under 16/8 h light/dark cycle at 25 ± 2 °C and 130μmolm^−2^ s^−1^ illumination intensity. After tissue culture of 30 days, well-growing plants were cultured in the rooting medium for 45 days. On the one hand, more than 200 diploids and more than 200 autotetraploid plants with uniform growth were transferred to 3% Hoagland and Arnon solution [93] (pH 6.0 ± 0.2) with dark for 3 days and light for 7 days before treatment. The nutrient solution was replaced every 2 days and kept sterile. Following PEG6000 was gradually applied to the seedlings to avoid osmotic shock which is 5%, 10%, 15% and finally 20% over 1-day intervals. All tissue samples were measured with five plants per replication and three biological replications per sample. Among them, DCK and TCK, D6h and T6h, D12h and T12h, D48h and T48h were snap-frozen in liquid nitrogen and stored at − 80 °C for transcriptome sequencing and qRT-PCR verification. Furthermore, 2-month-old diploids and the autotetraploids were transplanted into soil, containing a mixture of turfy soil and vermiculite and grown in a greenhouse at a temperature of 24 ± 1 °C under a 16 h photoperiod with a 3klx intensity of cool white fluorescent light. When plants growth was stable, drought stress was performed. After watering through, not watered until the soil water content reaches 4% and held for 7 days to determine the drought tolerance phenotype of diploid sour jujube and its autotetraploid.

### Physiological measurements

In both the diploid and the autotetraploid, eight physiological traits were measured under the increased drought stress intensity. Chlorophyll contents were measured with SPAD-502 Plus chlorophyll meter (Konica Minolta, Japan) from 14 plants with three technical repetitions. Other seven physiological traits were detected from five different plants and three biological repetitions in each sample with NanoQuant plate reader (Tecan, Switzerland). The SS contents were determined by sulfuric acid anthrone colorimetric method at 620 nm [94]. For SP extraction, 0.2 g samples were put into liquid nitrogen to grind in powder form. After adding 5 ml phosphate buffer (PBS, 0.05 M, pH 7.0), samples were centrifugated for 15 min at 5000 rpm at 4 °C and the supernatants were used for measurements of SP, MDA, SOD, POD, CAT using corresponding assay. The methods of Coomassie Brilliant Blue G-250 staining [95] were used to detect the content of SP at 595 nm with the standard curve drawn by bovine serum albumin in advance. For the activity of SOD, POD and CAT, after adding corresponding reagent, these traits were respectively calculated at 560 nm, 470 nm and 240 nm [96]. For MDA content, thiobarbituric acid was used as reaction mixture to detect at 600 nm and 532 nm respectively [97]. The proline contents were measured with the method of Acidic-ninhydrin at 520 nm [98]. In addition, for O^2−^ level, leaves sampled at 6 h, 12 h and 48 h under drought condition were put into 1 mg/ml nitroblue tetrazolium to stain for 12 h in dark, 28 °C and following were faded with absolute ethyl alcohol.

Excel 2010 and SPSS (version 19.0) were used to analyze the data through one-way analysis of variance. Duncan’s multiple range test was used for multiple comparisons.

### RNA sequencing, mapping and annotation

Leaves under control and drought treatment of 6 h, 12 h and 48 h were sampled from diploid and the autotetraploid for RNA-seq. Total RNA was extracted from 100 mg sample with Plant RNA Kit (Omega Biotech, USA) and purified with RNase-Free DNase set. Following, cDNA library was constructed and the library concentration and insert size was respectively assessed on the Qubit2.0 (Invitrogen, USA) and Agilent Bioanalyzer 2100 system, and then the effective concentration of the library was accurately quantified through Q-PCR. Prepared high quality libraries were sequenced on Illumina HiSeq X-ten platform to generate paired-end raw reads.

After filtering all adapter- and poly-N containing sequences and low-quality reads, clean reads were used to calculated base quality score Q20 and Q30 values and base content distribution. Clean data was aligned to “Dongzao” jujube reference genome sequence using HISAT2 software and alignment rate was calculated [99, 100].

Mapped reads were matched and compared to genome annotation information using StringTie software [101]. To supplement original genome annotation information, new jujube transcripts and genes were explored and analyzed. Blast software was used to map new genes to NR [102], Swiss-Prot [103], GO [104], COG [105], KOG [106], Pfam [107], KEGG [108] databases. KEGG pathway enrichment analysis was performed using KOBAS (version 2.0) [109].

### Gene expression quantification and differential expression analysis

The mapped reads number and transcript length of samples were normalized. Through the maximum flow algorithm in StringTie, Fragments per kilobase per million mapped reads (FPKM value) [110] were introduced as an indicator to measure gene expression level. Biological repeat correlation was assessed by Pearson's Correlation Coefficient [111].

EdgeR [112] was conducted for differential expression analysis. The resulting *P*-values were adjusted using Benjamini and Hochberg’s approach for controlling the False Discovery Rate (FDR). In comparison groups of D6h vs. DCK, D12h vs. DCK, D48h vs. DCK and T6h vs. TCK, T12h vs. TCK, T48h vs. TCK, genes meeting screening criteria, Fold Change ≥ 2 and FDR ≤ 0.05, were considered as DEGs, while in D6h vs. T6h, D12h vs. T12 and D48h vs. T48h, criteria was set as Fold Change ≥ 1.5 and FDR ≤ 0.05.

### Gene annotation and functional analyses

To understand gene functions, identified DEGs were annotated to GO database, classified into three parts: molecular function, biological process, and cellular component categories and were annotated against KEGG and Swiss-Prot. The plantTFDB [113] were used to identify genes coding for TFs among the DEGs.

To identify the potential hub genes underlying the differential responses of two sour jujube materials under drought stress, WGCNA was established through R package. As shown in Additional file 1: Figure S5, up-regulated genes that encoding TF identified in DCK vs. TCK, D6h vs. T6h, D12h vs. T12 and D48h vs. T48h were regarded as baits. Genes that showed a twofold difference (FDR ≤ 0.05) compared to the corresponding ‘0’ (untreated) point at least at one time point in diploid and the autotetraploid were considered as the pool. The genes with kME > 0.7 were selected as the members of the module. After computing the correlation between modules and baits, the most relevant three modules of up-regulated TF genes with r < − 0.3 or > 0.4 and *p* < 0.01 was selected to analyze. The hub genes were further screened by expression correlation with other genes in the module, namely, connectivity. Gene co-expression networks were visual with Cytoscape (version 3.8.2).

### Validation of DEGs and screening of candidate genes by qRT-PCR analysis

Total RNAs were extracted from leaves of control and treatment groups as described above. After determining RNA concentration and integrity, first-strand cDNA was reverse transcribed using a FastKing RT kit (TIANGEN, China) according to the manufacturer’s instructions. Total 28 DEGs including at least one differentially expressed comparison were selected for qRT-PCR analysis. Primers in this experiment designed using Beacon Designer (version 7.7) were listed in Additional file 2: Table S10. The real time PCR amplifications were carried out with 2 × SYBR® Green qPCR Mix Kit (Aidlab, China) in 25 μl volume on the 7500 Fast Real-Time instrument (Thermo Fisher, Singapore) using the following cycling protocol: 3 min at 94 °C, followed by 40 cycles for 20 s at 94 °C, 20 s at 55 °C, and 30 s at 72 °C (signal acquisition at 72 °C). The 2^−ΔΔCt^ method was used to calculate DEGs relative expression level [114].

### Statistical analysis

Statistical data, shown as the means ± SE (standard error), were processed with Microsoft Office Excel. Statistical differences of physiology were determined based on a t-test with *p < 0.05 and **p < 0.01 as significant. The one-way analysis of variance (ANOVA) method in SPSS (IBM, NY, USA) was used to analyze ΔCt value with a significance level of 0.05 and the differences were compared by LSD multiple range test.

## Supplementary Information

**Additional file 1: Figure S1.** PCA analysis of all 24 diploid and autotetraploid samples. **Figure S2.** Quantitative real time PCR analysis of selected DEGs involved in KEGG enrichment pathways from diploid and autotetraploid. **Figure S3.** qRT-PCR analysis of autotetraploid-specific ROS scavenging-related DEGs in diploid and the autotetraploid. **Figure S4.** Module-trait relationships in diploid and autotetraploid under drought stress conditions. **Figure S5.** The hub genes screening process.

**Additional file 2: Table S1.** Statistics of all samples transcriptome sequencing. **Table S2.** Statistics of alignment rates to the reference genome for each sample. **Table S3.** List of DEGs identified after drought treatment in diploid and autotetraploid sour jujube. **Table S4.** List of DEGs identified between diploid and autotetraploid sour jujube after drought treatment. **Table S5.** Differential expression levels of 83 DEGs in three pathways, anthocyanin synthesis, glutathione metabolism and plant hormone signal transduction. **Table S6.** Common DEGs involved in peroxisome function in diploid and the autotetraploid after drought treatment. **Table S7.** Traits for WGCNA analysis. **Table S8.** Pool for WGCNA analysis. **Table S9.** Statistics of genes in each module. **Table S10.** Primers for quantitative real time PCR.

## Data Availability

The datasets supporting the conclusions of this article are included within the article and its additional files.

## Abbreviations

DEGs: Differentially expressed genes
TFs: Transcription factors
SS: Soluble sugar
SP: Soluble protein
SOD: Superoxide dismutase
POD: Peroxidase
CAT: Catalase
MDA: Malonaldehyde
