# Supplementary material for: Multiple responses contribute to the enhanced drought tolerance of the autotetraploid Ziziphus jujuba Mill. var. spinosa
Source: Cell Biosci. 2021 Jun 30;11:119. doi: 10.1186/s13578-021-00633-1 (PMC8243571; doi:10.1186/s13578-021-00633-1)
Supplement: Supplementary file 1 — Additional file 1: Figure S1. PCA analysis of all 24 diploid and autotetraploid samples. Figure S2. Quantitative real time PCR analysis of selected DEGs involved in KEGG enrichment pathways from diploid and autotetraploid. Figure S3. qRT-PCR analysis of autotetraploid-specific ROS scavenging-related DEGs in diploid and the autotetraploid. Figure S4. Module-trait relationships in diploid and autotetraploid under drought stress conditions. Figure S5. The hub genes screening process. [file 13578_2021_633_MOESM1_ESM.doc]

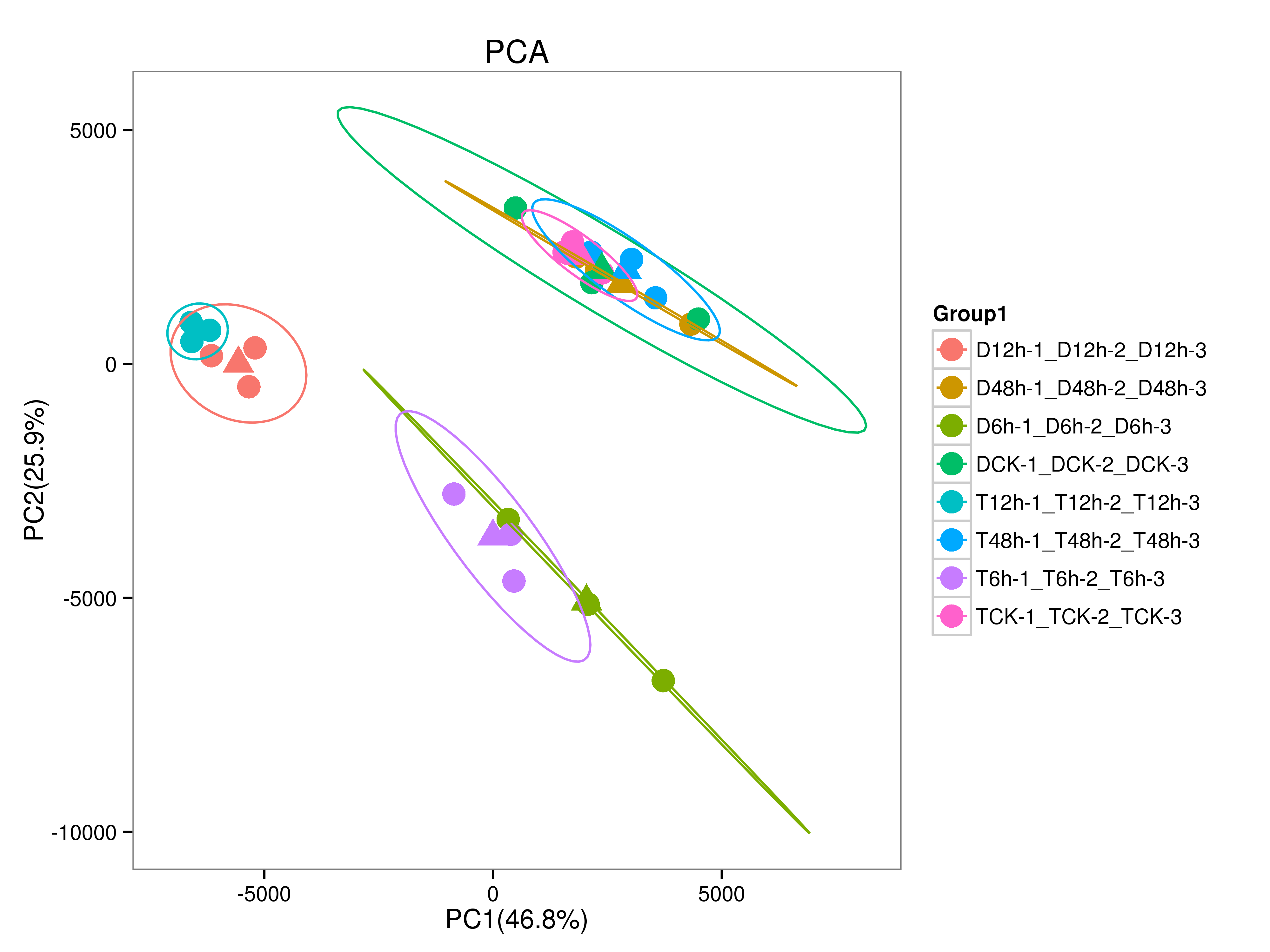


**Figure S1** PCA analysis of all 24 diploid and autotetraploid samples. DCK: diploid under control condition; TCK: autotetraploid under control condition; D6h, D12h, D48h: diploid under 6h, 12h, 48h of drought stress; T6h, T12h, T48h: autotetraploid under 6h, 12h, 48h of drought stress. '1, 2, 3' indicate three repeats.


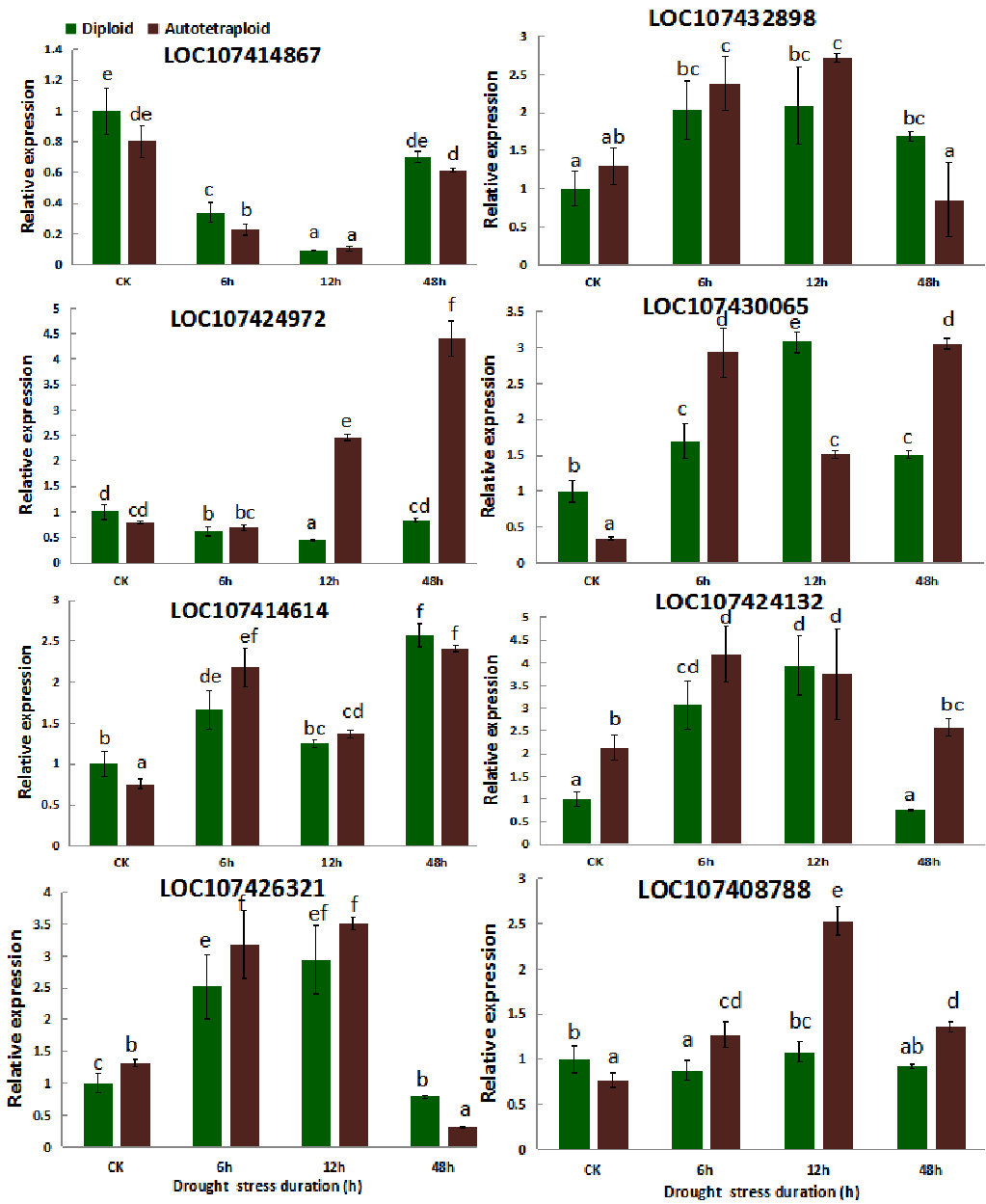


**Figure S2** Quatative real time PCR analysis of selected DEGs involved in KEGG enrichment pathways from diploid and autotetraploid. The vertical bars show the standard deviation and a significance level of 0.05 was used for different letters above bars.


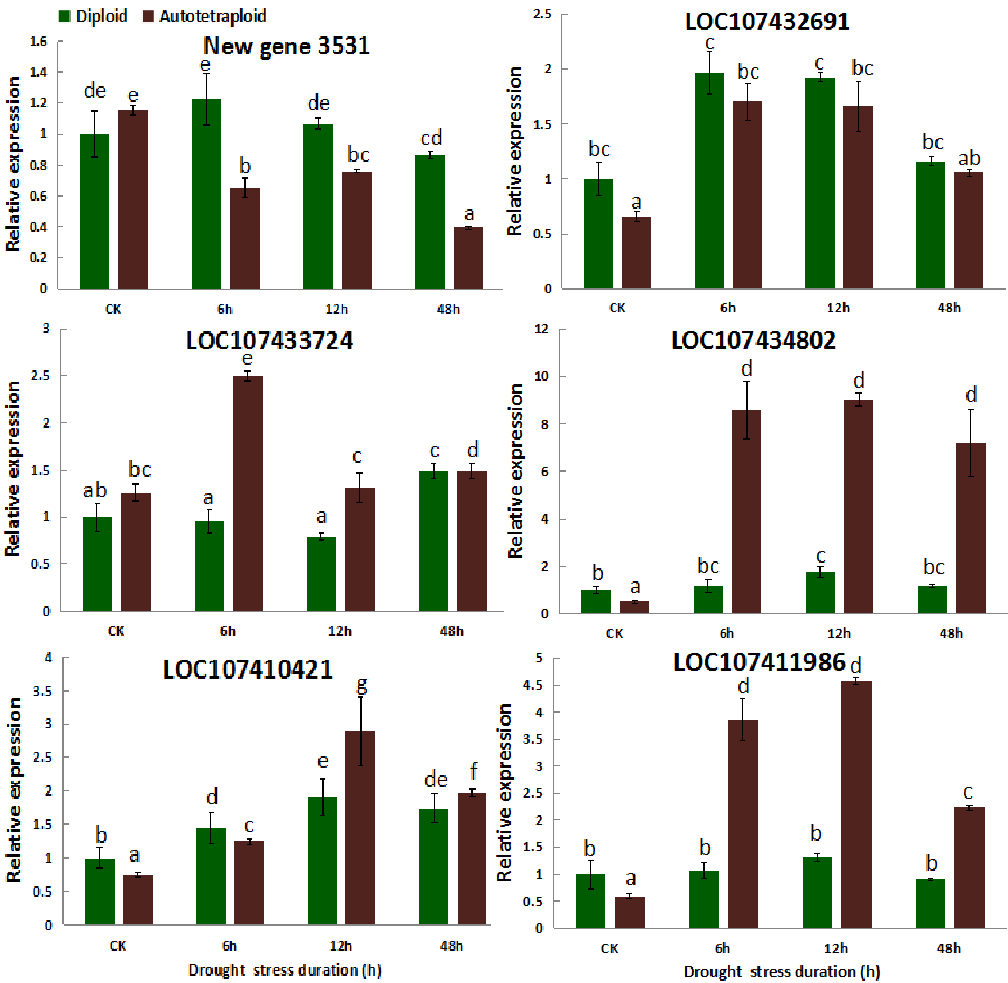


**Figure S3** qRT-PCR analysis of autotetraploid-specific ROS scavenging-related DEGs in diploid and the autotetraploid. The vertical bars show the standard deviation and a significance level of 0.05 was used for different letters above bars.


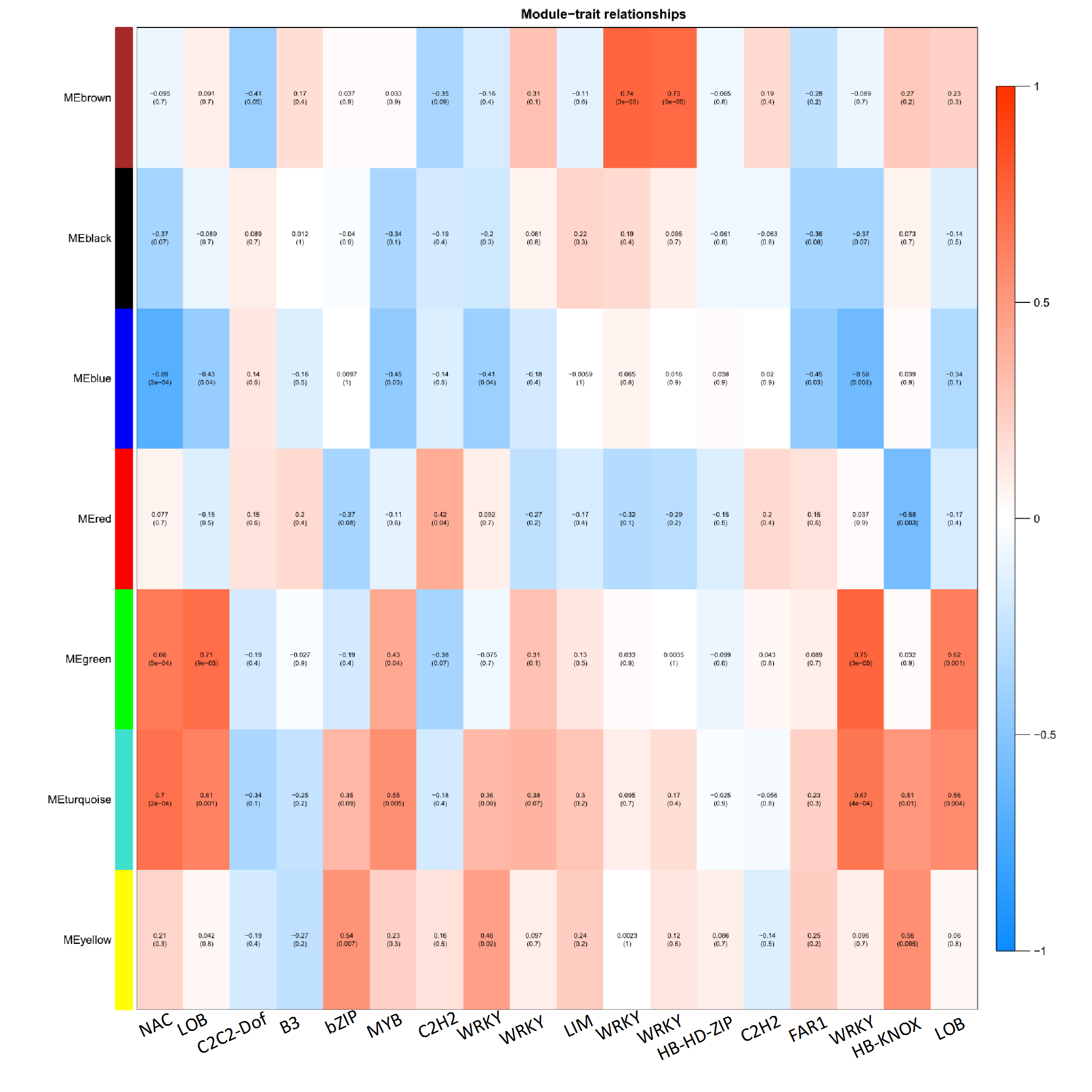


**Figure S4** Module-trait relationships in diploid and autotetraploid under drought stress conditions. The x-axis represents the traits and the y-axis is the module. The first value in each box is the correlation value, and the *p* value in brackets. The positive and negative values represent positive and negative correlation. Legend, 0 represents no correlation, -1 represents the greatest negative correlation, and 1 represents the greatest positive correlation.


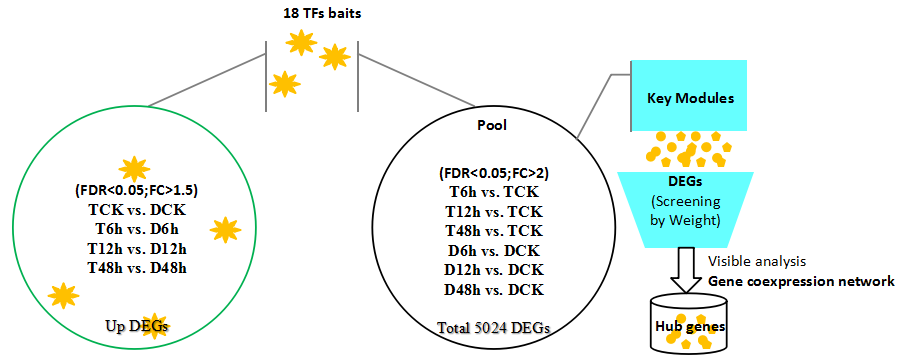


**Figure S5** The hub genes screening process. The circles represent the unions of comparison groups. The yellow stars in the green circle represent the bait, and the gray circle represents the pool.
